# Exploring simple triplet representation learning^☆^

**DOI:** 10.1016/j.csbj.2024.04.004

**Published:** 2024-04-12

**Authors:** Zeyu Ren, Quan Lan, Yudong Zhang, Shuihua Wang

**Affiliations:** a University of Leicester, Leicester, UK; b Department of Neurology, First Affiliated Hospital of Xiamen University, China; c Department of Information Technology, Faculty of Computing and Information Technology, King Abdulaziz University, Jeddah 21589, Saudi Arabia; d Department of Biological Sciences, Xi'an Jiaotong-Liverpool University, Suzhou, Jiangsu 215123, China; e Department of Mathematical Sciences, University of Liverpool, Liverpool, L69 3BX, UK

**Keywords:** Deep learning, Self-supervised learning, Medical image analysis, Semi-supervised learning, Contrastive learning, Machine learning

## Abstract

Fully supervised learning methods necessitate a substantial volume of labelled training instances, a process that is typically both labour-intensive and costly. In the realm of medical image analysis, this issue is further amplified, as annotated medical images are considerably more scarce than their unlabelled counterparts. Consequently, leveraging unlabelled images to extract meaningful underlying knowledge presents a formidable challenge in medical image analysis. This paper introduces a simple triple-view unsupervised representation learning model (SimTrip) combined with a triple-view architecture and loss function, aiming to learn meaningful inherent knowledge efficiently from unlabelled data with small batch size. With the meaningful representation extracted from unlabelled data, our model demonstrates exemplary performance across two medical image datasets. It achieves this using only partial labels and outperforms other state-of-the-art methods. The method we present herein offers a novel paradigm for unsupervised representation learning, establishing a baseline that is poised to inspire the development of more intricate SimTrip-based methods across a spectrum of computer vision applications. Code and user guide are released at https://github.com/JerryRollingUp/SimTripSystem, the system also runs at http://43.131.9.159:5000/.

## Graphical abstract

**Figure:**
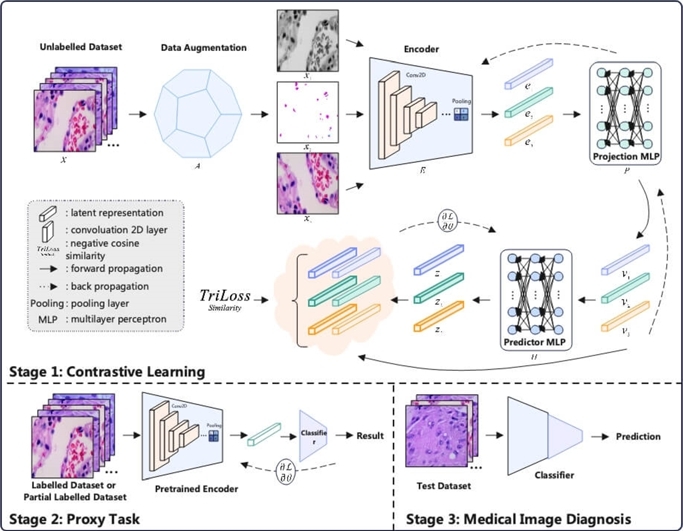

## 1 Introduction

To achieve superior performance, fully supervised learning methods require substantial quantities of labelled data. Nonetheless, acquiring the ground-truth labels for unannotated images proves to be both challenging and costly, particularly in medical image analysis, annotators are required to possess specialized knowledge and adhere to more meticulous labelling processes in comparison to other domains. Recently, self-supervised visual representation learning has emerged as a viable solution to address the scarcity of labelled datasets in medical image analysis. Moreover, self-supervised learning-based methods can get the same or superior performance than the fully supervised learning-based methods [1]. Utilizing self-supervised learning, we can generate a representation from the unlabelled dataset, then transfer the representation as an input to the proxy tasks such as linear classifier, fine-tuning, and transfer learning, which necessitate fewer labelled images to train the model. This approach enables efficient outperformance over other fully supervised learning-based methods across various downstream tasks.

Over the past decade, self-supervised learning has gained much attention, initially emerging within the robotics domain. Its primary objective is extracting valuable mutual representations from unlabelled data, thereby benefiting the downstream tasks [2]. Recently, contrastive learning, a subset of self-supervised learning, has demonstrated its exceptional performance in various downstream tasks [3], [4], [5]. Lots of studies prove that utilizing the well-trained contrastive learning model on the target domain can achieve the same or higher performance than fully supervised learning. Among these studies, SimCLR [6] stands out as a pivotal work in contrastive learning. It constructs positive and negative sample pairs, maximizes similarity with positive pairs, and minimizes differences with negative pairs. Following this, MoCo [7] introduces a momentum encoder and a dynamic dictionary equipped with a queue for storing negative samples. Subsequently, BYOL [8] proposes the innovative idea of discarding negative samples to construct the contrastive learning-based method. Building upon this concept, SimSiam [9] further refines the approach by eliminating the momentum encoder in BYOL. This results in a Siamese architecture capable of efficiently extracting mutual knowledge from the unlabelled dataset.

While self-supervised learning achieved remarkable success in many computer vision tasks, there are also some challenges that need to be solved, including model collapse, extensive computational resource requirements, large batch sizes and meaningless underlying representations for downstream tasks. Model collapse is a scenario where all outputs of the model converge to a constant, resulting in identical feature maps regardless of the training method employed, essentially leading to a trivial solution. Several strategies have been proposed to mitigate model collapse. In SimCLR [6], the negative pairs serve to avoid trivial solutions in the solution space. Clustering [10] clusters representations alternately and tries to predict the cluster assignment to avoid model collapse. Moreover, BYOL [8] employs a momentum encoder with positive pairs to circumvent trivial solutions. Most self-supervised methods still demand extensive computational resources and large batch sizes. For instance, SimCLR [6] trains on 128 TPU v3 cores with a batch size of 4096. The SwAV [11] also needs batch size from 256 to 4096 with 64 V-100 GPUs. In practical scenarios, particularly in medical image analysis with prevalent high-resolution images, these requirements render the training process challenging and costly. Hence, there is an evident need for developing self-supervised methodologies with smaller batch sizes and reduced computational resources.

**Fig. 1:**
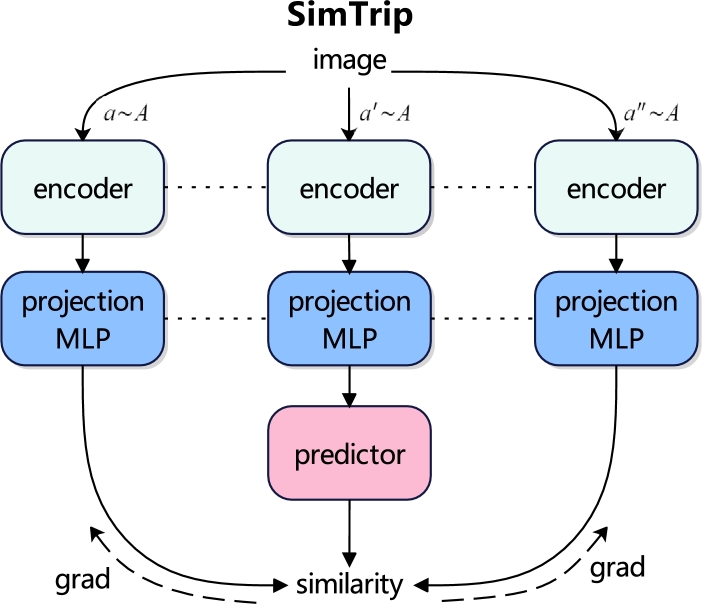
SimTrip architecture, includes an encoder, projection and prediction multilayer perceptron (MLP). The terms *a*, *a*′, and *a*″ represent a specific data augmentation method applied, while *A* encompasses all the data augmentation methods utilized within the SimTrip. The dotted line indicates that the connected components share the same architecture and weights, and the dotted line with the arrow accompanied by ‘grad’ implies a stop-gradient.

In this work, we introduce SimTrip, a novel self-supervised learning method capable of learning meaningful representations efficiently with smaller batch sizes and reduced computational power. SimTrip draws inspiration from the conventional triplet network [12], which typically comprises three inputs: an anchor, a positive and a negative input, with the loss calculated across the outputs of these inputs. However, unlike the triplet network, SimTrip utilizes three distinct augmented views derived from a single image. An illustrative overview of SimTrip architecture is presented in Fig. 1. In conjunction with this architecture, we proposed a novel loss function TriLoss to compute the loss between different branches of SimTrip. Our evaluation of SimTrip across two medical image datasets with two proxy tasks: linear evaluation and fine-tuning, also supplemented by an ablation study, demonstrates that our method can efficiently extract underlying representations with small batch size and reduced computational power.

This study makes the following key contributions:

• We present a novel paradigm for unsupervised representation learning, efficiently circumventing the model collapse problem with triple-view loss function TriLoss.
• Our model enables the learning of meaningful inherent knowledge with triple-view architecture, it directly maximizes the similarity of one image's triple views instead of (*i*) negative sample pairs, (ii) large batch size, and (iii) momentum encoders.
• Our model can be combined with other models to implement different proxy tasks, including linear classification, fine-tuning, and transfer learning. The results of our evaluation on linear evaluation and fine-tuning tasks outperform other state-of-the-art methods.
• The model can efficiently extract underlying representations with a small batch size on lower computational power.

## 2 Related work

**Self-Supervised Learning:** Contrary to supervised learning, which is constrained by the quantity of labelled data, self-supervised learning excels in extracting meaningful underlying knowledge from vast amounts of unlabelled data [2], [6]. This paradigm shift has catalyzed significant advancements in the field of computer vision with large datasets, exemplified by models like SEER, a model trained on an extensive dataset comprising 1 billion images [19]. Within computer vision, self-supervised learning methods have showcased remarkable efficacy, achieving performance levels comparable to, or even surpassing, those of supervised models trained on labelled datasets, as evidenced in challenging benchmarks like ImageNet [7], [20]. Beyond computer vision, self-supervised learning has demonstrated its versatility and effectiveness across various modalities, including audio, video, and time series data [21], [22], [23].

Self-supervised learning introduces a pretext task utilizing unlabelled data to generate meaningful representations, subsequently enabling the application of various downstream tasks. In the realm of computer vision, these extracted representations are used in classification, segmentation, object detection, and more. Here, we categorize self-supervised learning-based methods as follows [24]:

• **Information Restoration:** This approach aims to reconstruct missing information in an image. It is achieved by initially masking or removing a specific part of the image, followed by a model to restore the image. Notable works in this category include colourization [25] and image inpainting [26].
• **Using Temporal Relationships in Video:** By pre-training on videos, self-supervised learning can yield single-image representations, which can then be employed in various downstream tasks. An example of this approach is the work by Wang et al. [27], where a model is pre-trained with videos to compute similarities in representations of the same target across two different frames, resulting in enhanced performance in object detection.
• **Learning Spatial Context:** This category encompasses works focused on training models to understand spatial relationships and orientations among objects within a scene. A prominent example is the method proposed by Doersch et al. [28], which predicts the relative location of two patches randomly selected from different locations of the image.
• **Grouping Similar Images Together:** This method involves clustering similar images by comparing their semantic information. A classic technique widely employed in a self-supervised manner is K-means clustering [10].
• **Generative Models:** Generative models aim to learn the underlying patterns of data and then generate new, similar data. They can be trained to generate diverse modalities, including images, texts, or audio and videos. Representative works in this category include denoising autoencoders [29], and generative adversarial networks (GANs) [30].
• **Multi-View Invariance:** Many self-supervised methods aim to produce various representations under different transforms. Contrastive learning, in particular, encourages models to generate diverse augmented representations from the same input. Prior to contrastive learning, numerous works sought to enforce invariance in representations through different methodologies, such as pseudo-labelling [31] and virtual adversarial training [32].

**Contrastive Learning:** Contrastive learning represents a discriminative approach, aiming to draw similar data points closer together while distancing dissimilar ones [13]. Specifically, it employs a similarity metric to access the representations produced by the encoder and then calculate the loss between varying representations. Recently, contrastive learning has emerged as a predominant method within the domain of self-supervised learning, garnering substantial attention and application [3], [6], [7], [8], [14], [15], [16], [17], [18].

Among these contributions, contrastive learning derives significant advantages from the construction of positive and negative pairs [5], [6], [7], [18]. These pairs can be retained in a memory bank [5], necessitating considerable memory capacity to store all potential pairings. SimCLR [6] addresses this challenge by forming positive and negative pairs within a batch, necessitating a large batch size for effective model training. On the other hand, the Siamese network-based MoCo [7] introduces a queue for storing negative samples and employs a momentum encoder for model training. In contrast, SimSiam [9] utilizes a Siamese network without the momentum encoder, requiring only positive pairs during the training phase. BYOL [8] follows a similar approach, eliminating the need for negative pairs but incorporating a momentum encoder, distinguishing it from SimSiam.

## 3 Methodology

### 3.1 Description of SimTrip

**Fig. 2:**
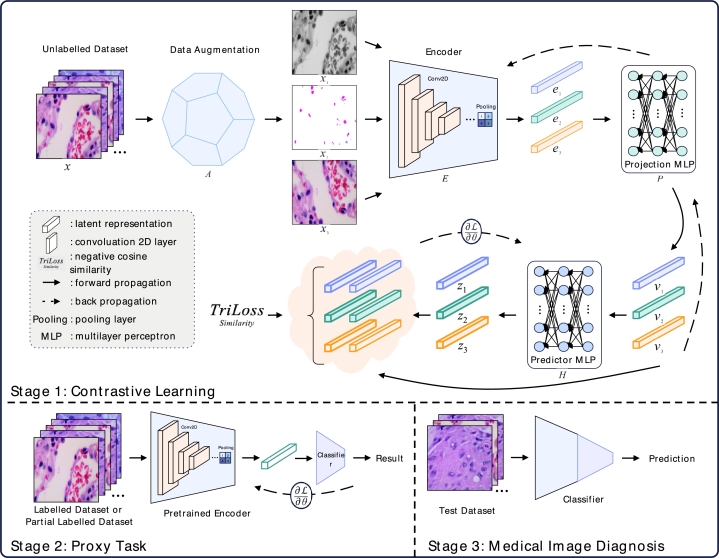
The pipeline of SimTrip, including three stages. The first stage is contrastive learning, where three representations, *x*_1_, *x*_2_, *x*_3_, are generated via a data augmentation module. Following an encoder that uses ResNet50 as the backbone to process these representations. The three resulting vectors are then passed through a projection and a prediction MLP, with TriLoss employed to compute the loss across three branches. In the second stage, the projection and prediction MLP are omitted, and the pre-trained encoder from the first stage is utilized for proxy tasks, including linear evaluation, transfer learning, fine-tuning and others. Finally, the third stage involves implementing medical image diagnosis using the classifier trained in the second stage.

The entire pipeline of SimTrip is delineated in Fig. 2. Drawing inspiration from the premise that the incorporation of suitable augmented views can enhance mutual information extraction and foster a robust representation across various transformations, especially when the model trains with smaller batch size [18]. We introduce a triplet network architecture with three augmented views complemented by a triple-view loss function TriLoss. The architecture is different from the Siamese architecture employed by SimSiam [9] and BYOL [8]. Moreover, unlike the standard triplet network, which utilizes anchor, positive and negative samples, SimTrip comprises three distinct augmented views derived from a single image. Specifically, our triplet network features three parallel branches encompassing an augmentation module, an encoder, a projection MLP and a prediction MLP with the same structure and weights. The triplet loss is then computed based on the outputs from three branches.

As depicted in Fig. 2, our model introduces input *x* into a data augmentation module *A*, yielding three augmented views: $x_{1}$, $x_{2}$, and $x_{3}$. These views are then fed into an encoder *E*, producing three vectors: $e_{1}$, $e_{2}$ and $e_{3}$. We employ ResNet50 [33] as the backbone for the encoder, which can be substituted with alternative deep learning architectures if necessary. The encoder is coupled with a projection MLP *P*, generating three vectors: $v_{1}$, $v_{2}$ and $v_{3}$. The computations of $v_{1}$, $v_{2}$ and $v_{3}$ are delineated in Eq. (1), where $v_{i}$ denotes the vectors from projection MLP from different branches with corresponding view $x_{j}$.

(1) $$v_{i}≜P(E(x_{j})).$$

A prediction MLP *H* is also incorporated subsequent to the projection MLP, producing vectors $z_{1}$, $z_{2}$, and $z_{3}$ as described by Eq. (2). It is important to note that the encoder, projection and prediction MLP across three branches are identical in both weights and architecture.

(2) $$z_{i}≜H(P(E(x_{j}))).$$

Finally, to align one view with the others, we minimize the negative cosine similarity between these vectors, as delineated in Eq. (3):

(3) $$N\left(v_{i},z_{j}\right)=-\frac{v_{i}}{{\left\|v_{i}\right\|}_{2}}\cdot \frac{z_{j}}{{\left\|z_{j}\right\|}_{2}},$$

where ‖⋅|| denotes the $l_{2}$-norm, which is equivalent to the mean squared error of $l_{2}$-normalized vectors [8]. Consequently, we define a triplet loss following the framework outlined in [8], formalized as Eq. (4). This equation characterizes the loss associated with a single image, while the aggregate loss is computed by averaging the individual losses across all images. The minimum value of this loss function is -1.

(4) $$L=\frac{1}{2}N\left(z_{1},v_{2}\right)+\frac{1}{2}N\left(z_{2},v_{1}\right)+\frac{1}{2}N\left(z_{1},v_{3}\right)+\frac{1}{2}N\left(z_{3},v_{1}\right)+\frac{1}{2}N\left(z_{2},v_{3}\right)+\frac{1}{2}N\left(z_{3},v_{2}\right).$$

Applying the $l_{2}$-norm, negative cosine similarity to Eq. (4), we get the loss function shown in Eq. (5):

(5) $$L=\frac{1}{2}\left(-\frac{z_{1}}{\sqrt{\sum z_{1i}^{2}}}\cdot \frac{v_{2}}{\sqrt{\sum v_{2i}^{2}}}\right)+\frac{1}{2}\left(-\frac{z_{2}}{\sqrt{\sum z_{2i}^{2}}}\cdot \frac{v_{1}}{\sqrt{\sum v_{1i}^{2}}}\right)+\frac{1}{2}\left(-\frac{z_{1}}{\sqrt{\sum z_{1i}^{2}}}\cdot \frac{v_{3}}{\sqrt{\sum v_{3i}^{2}}}\right)+\frac{1}{2}\left(-\frac{z_{3}}{\sqrt{\sum z_{3i}^{2}}}\cdot \frac{v_{1}}{\sqrt{\sum v_{1i}^{2}}}\right)+\frac{1}{2}\left(-\frac{z_{2}}{\sqrt{\sum z_{2i}^{2}}}\cdot \frac{v_{3}}{\sqrt{\sum v_{3i}^{2}}}\right)+\frac{1}{2}\left(-\frac{z_{3}}{\sqrt{\sum z_{3i}^{2}}}\cdot \frac{v_{2}}{\sqrt{\sum v_{2i}^{2}}}\right),$$

where $z_{1i}$, $z_{2i}$, $z_{3i}$, $v_{1i}$, $v_{2i}$, and $v_{3i}$ represent the individual components of the vectors $z_{1}$, $z_{2}$, $z_{3}$, $v_{1}$, $v_{2}$, and $v_{3}$ respectively. The summations under the square roots calculate the square of each component, and their sum is then square-rooted to find the $l_{2}$-norm of each vector.

An important element in our SimTrip is the incorporation of the stop-gradient operation, as illustrated in Fig. 2, the implementation of stop-gradient by the modification detailed in Eqn. (3):

(6) $$N\left(z_{j},\text{ stopgrad}\left(v_{i}\right)\right).$$

In Eqn. (6), the $z_{j}$ is treated as a constant and the inclusion of the stop-gradient operation within the loss function in Eqn. (4) yields the Eq. (7):

(7) $$L=\frac{1}{2}N\left(z_{1},\text{ stopgrad}\left(v_{2}\right)\right)+\frac{1}{2}N\left(z_{2},\text{ stopgrad}\left(v_{1}\right)\right)+\frac{1}{2}N\left(z_{1},\text{ stopgrad}\left(v_{3}\right)\right)+\frac{1}{2}N\left(z_{3},\text{ stopgrad}\left(v_{1}\right)\right)+\frac{1}{2}N\left(z_{2},\text{ stopgrad}\left(v_{3}\right)\right)+\frac{1}{2}N\left(z_{3},\text{ stopgrad}\left(v_{2}\right)\right).$$

The algorithm of SimTrip is shown in Algorithm 1.

**Algorithm 1:**
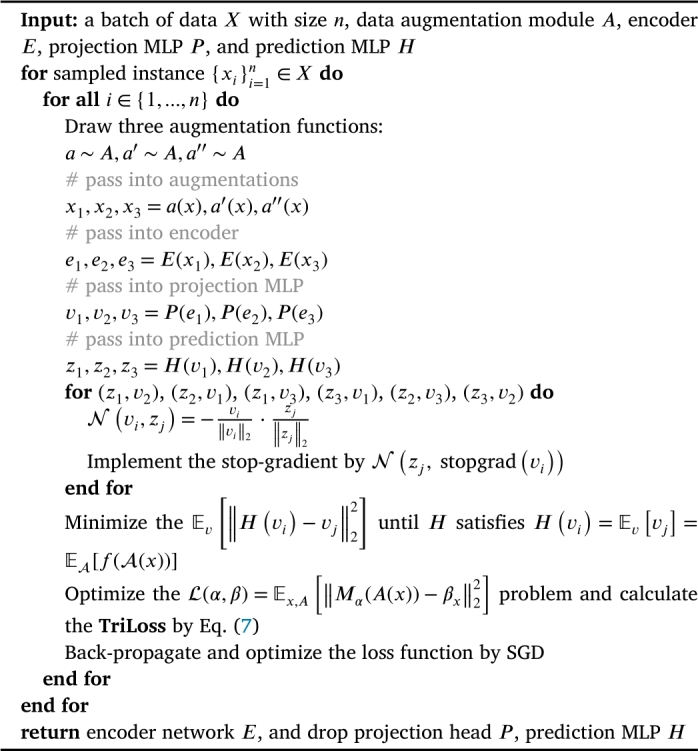
The algorithm of SimTrip.

We theorize that the SimTrip operate similarly to an Expectation-Maximization (EM) like algorithm. It subtly incorporates two variable sets and addresses two inherent sub-problems. The inclusion of the stop-gradient results in the additional variable set. The loss function can also be considered in the following form:

(8) $$L(\alpha ,\beta )=E_{x,A}\left[{\left\|M_{\alpha }(A(x))-\beta_{x}\right\|}_{2}^{2}\right],$$

where *x* is an image, *A* is the data augmentation, $M_{\alpha }$ represents a model with parameter *α*, the additional variable set denotes as *β*, and e[⋅] pertains to the distribution of images and their augmentations.

As shown in Eq. (8), we incorporate an additional set of variables, represented as *β*. Here, the total image count is the size of *β*, $\beta_{x}$ is the representation of an image *x*, with the subscript *x* indicating the use of the image index to retrieve a sub-vector of *β*. It is worth noting that *β* might not be a direct output from a neural network. Given this setup, our focus turns to resolving:

(9) $$\min_{\alpha ,\beta }E_{x,A}\left[{\left\|M_{\alpha }(A(x))-\beta_{x}\right\|}_{2}^{2}\right].$$

This problem depicted in Eq. (9) is similar to k-means clustering [55], where the *α* denotes the trainable parameters of the encoder, and $\beta_{x}$ is akin to the allocation vector of the instance *x*.

Eq. (9) also can be tackled using an alternative algorithm, where one variable set is held constant while solving for the other. Then, it alternately involves the following two sub-issues:

(10) $$\alpha^{t}\leftarrow \arg\min_{\alpha }E_{x,A}\left[{\left\|M_{\alpha }(A(x))-\beta_{x}^{t-1}\right\|}_{2}^{2}\right],$$

(11) $$\beta^{t}\leftarrow \arg\min_{\beta }E_{x,A}\left[{\left\|M_{\alpha^{t}}(A(x))-\beta_{x}\right\|}_{2}^{2}\right],$$

where *t* is the index of alternation and assigning denotes by the ←. To solve first problem (10), we use stop-gradient operation since the gradient does not back-propagate to $\beta^{t-1}$, making it a fixed value in Eq. (10). For each $\beta^{x}$, the second subproblem can be tackled separately. The objective now is to reduce the value of Eq. (12) for each *x*.

(12) $$E_{A}\left[{\left\|M_{\alpha^{t}}(A(x))-\beta_{x}\right\|}_{2}^{2}\right].$$

It needs to be mentioned that the expectation is based on the augmentation distribution *A*. Owing to the mean squared error, this subproblem can be solved by:

(13) $$\beta_{x}^{t}\leftarrow E_{A}\left[M_{\alpha^{t}}(A(x))\right].$$

We can approximate the SimTrip algorithm by a one-step alternation between Eqn. (10) and (11). Initially, we randomly sample a data augmentation method, denoted as $A^{\prime }$, allowing us to disregard the $E_{A}[\cdot ]$, as shown in Eq. (14).

(14) $$\beta_{x}^{t}\leftarrow \left[M_{\alpha^{t}}(A^{\prime }(x))\right].$$

Combine the Eq. (14) with Eq. (10), we get:

(15) $$\alpha^{t+1}\leftarrow \arg\min_{\alpha }E_{x,A}\left[{\left\|M_{\alpha }(A(x))-M_{\alpha^{t}}(A^{\prime }(x))\right\|}_{2}^{2}\right].$$

In Eq. (15), $\alpha^{t}$ represents a constant, and $A^{\prime }$ is an augmentation method randomly selected from the set *A*. This equation formulates the SimTrip architecture. By minimizing the loss and implementing Eq. (15) with a single SGD step, we obtain the SimTrip algorithm, where the stop-gradient operation is naturally integrated within SimTrip algorithm.

Regarding the effect of the predictor *H*, it aims to minimize the following equation:

(16) $$E_{v}\left[{\left\|H\left(v_{i}\right)-v_{j}\right\|}_{2}^{2}\right].$$

Here, $v_{i}$ and $v_{j}$ are the vectors generated from two adjacent branches, yielding three pairs in total.

When *H* satisfies Eq. (17), we achieve the optimal solution for *H*:

(17) $$H\left(v_{i}\right)=E_{v}\left[v_{j}\right]=E_{A}[f(A(x))].$$

As mentioned in Eq. (14), we ignore the expectation $E_{A}[\cdot ]$. However, the presence of predictor *H* compensates for this gap. Calculating the expectation $E_{A}$ in practice is infeasible. Nevertheless, we can employ a neural network (e.g., the prediction MLP *H*) to learn the parameters necessary to predict this expectation. After several epochs of training, the random sampling $A^{\prime }$ becomes implicitly distributed within the weights of the predictor.

### 3.2 Implementation details

In this section, we list all the implementation details of each component of SimTrip, including image augmentations, encoder, optimizer, projection MLP, prediction MLP, and classifier.

As Algorithm 1 describes, we draw three random image augmentations from the data augmentation module *A*, including cropping, resizing, colour jittering, greyscaling, gaussian blur, horizontal flipping, and vertical flipping. Each operation below is performed sequentially to generate three views:

• Random cropping is applied with a scale ranging from 0.08 to 1.0 and an aspect ratio of $\frac{3}{4}\times \frac{H}{W}$, *H* is the height of the image, and *W* is the width of the image.
• Resizing the cropped image to a uniform resolution of 256×256.
• Color jittering with random brightness (0.32), random contrast (from 0.68 to 1.32), random saturation (from 0.68 to 1.32), random hue (0.02) and probability of 0.8.
• Grayscaling is applied with a probability of 0.2.
• Gaussian blur with a standard deviation (std) randomly chosen between 0.1 and 2.0.
• Horizontal and vertical flipping with a probability of 0.5.

**Fig. 3:**
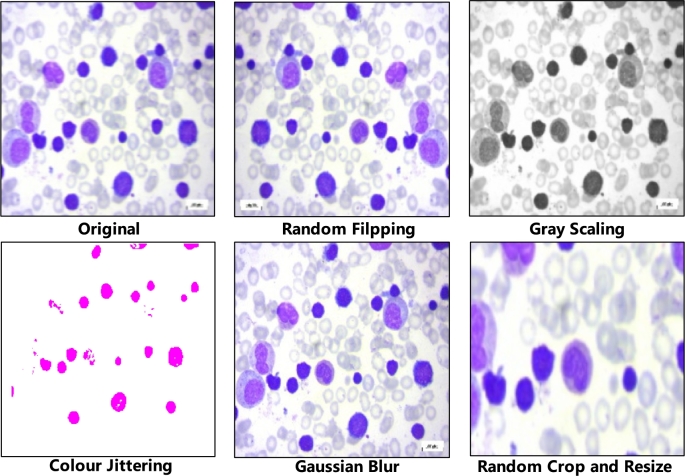
Augmented images by different data image augmentations, which include random flip, grey scaling, colour jittering, gaussian blur and random crop and resize (example from ALL dataset).

Fig. 3 illustrates the impact of these augmentations on a sample image from the ALL dataset.

**Fig. 4:**
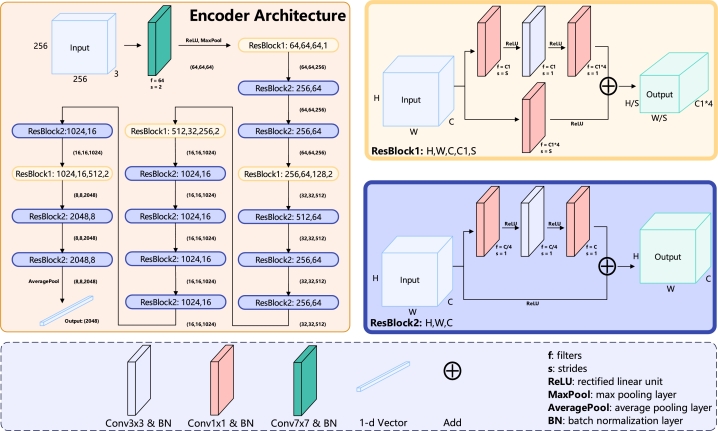
Encoder architecture. The encoder architecture utilizes ResNet50 with pre-trained ImageNet weights as its backbone. This architecture comprises two distinct residual blocks that connect layers with varying input and output sizes. In ResBlock1, the input and output sizes differ, whereas in ResBlock2, the input and output sizes are the same. Finally, an average pooling layer is added, which generates a 2048-dimensional vector for the projection MLP.

For the encoder, we use ResNet50 [33] as the default backbone. We also use the pre-trained weights of ImageNet for the ResNet50 during the training process, accompanied by a pooling layer to complete the encoder architecture. The details of the encoder architecture are shown in Fig. 4. It needs to be mentioned that in most of the representation learning methods, the ResNet50 is regarded as the default backbone of the encoder for the convenience of the comparison.

We use SGD optimizer in our method. Unlike other self-supervised methods [6], [8], [11] that require a large-batch optimizer such as LARS [34], SimTrip only needs a small batch size, and it also can perform well. The learning rate of the SGD optimizer starts from 0.05, following a cosine decay schedule [6], with a weight decay of 0.0005 and SGD momentum of 0.9.

**Fig. 5:**
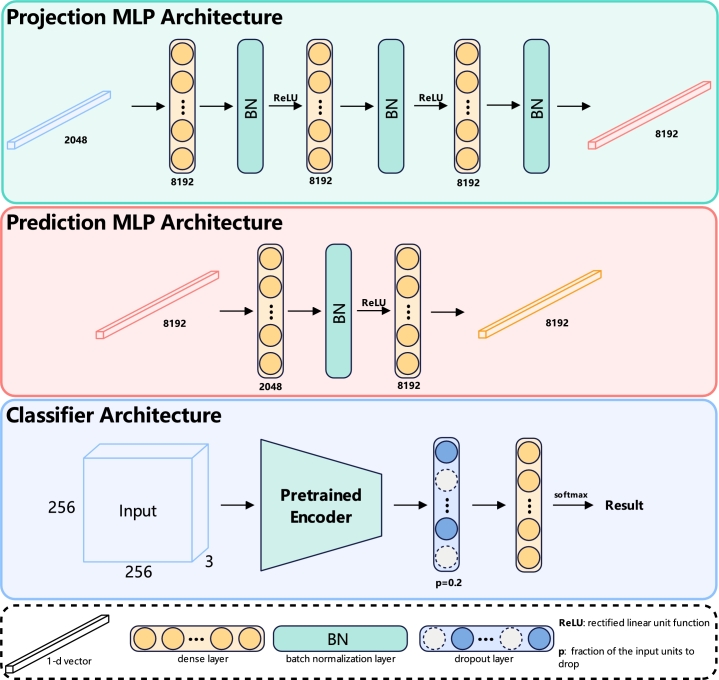
The architecture of projection MLP, prediction MLP and classifier. The projection MLP receives a 2048-dimensional vector output from the encoder and feeds it into three dense layers, each 8192-dimensional, accompanied by a batch normalization layer and ReLU function. However, the final dense layer does not connect to a ReLU function. This process results in an 8192-dimensional vector that is forwarded to the prediction MLP. The prediction MLP comprises a single 2048-dimensional dense layer, followed by a batch normalization layer and a ReLU function, and then it culminates in an 8192-dimensional dense layer that produces a corresponding vector. The classifier architecture integrates the pre-trained encoder with a dropout layer, set at a probability of 0.2, which then connects to a dense layer. The dimension of this dense layer corresponds to the number of classes in the dataset. Finally, a softmax function follows to compute the result.

The projection MLP (*P*) consists of three dense layers, each followed by a batch normalization (BN) layer and a ReLU activation function, except the last dense layer, which does not include a ReLU function. Each of these layers is 8192-dimensional. The details of the projection MLP architecture are shown in the Fig. 5.

The prediction MLP (*H*), structured as a bottleneck, has two dense layers. The first is connected to a BN layer and ReLU function, followed by the second dense layer. The input of the prediction MLP is 8192-dimensional, which is also the output of the projection MLP. The first dense layer is 2048-dimensional, and the second dense layer is 8192-dimensional. The details of the prediction MLP architecture are shown in the Fig. 5.

The classifier of the SimTrip takes the pre-trained encoder connected with a dropout layer and then uses a dense layer with softmax to predict the final output. The details of the classifier architecture are shown in the Fig. 5.

### 3.3 Method selection and comparison

**Fig. 6:**
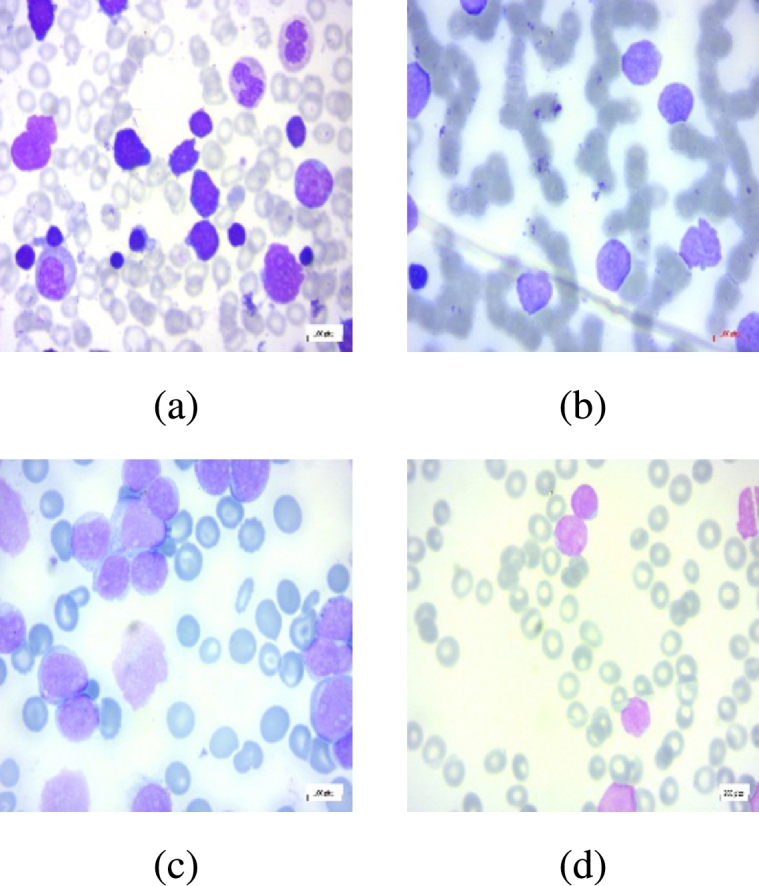
Comparison of Siamese architectures. The encoder consists of layers shared across both branches. The dashed lines represent the flow of gradient propagation. In BYOL, SwAV, and SimSiam, the absence of a dashed line suggests a stop-gradient. The symmetrization for these methods is not depicted for clarity. The elements highlighted in red are not present in SimTrip.

In this section, we compare the triplet architecture of SimTrip with other Siamese architectures, explaining how SimTrip can be viewed as an enhanced version of these existing methods by integrating them and introducing modifications. Fig. 6 abstracts these methods. It needs to be mentioned that the ‘encoder’ in Fig. 6 subsumes all the layers that can be shared between different branches, such as backbone and projection MLP. The red components in this figure are the missing parts of the SimTrip.

**Relation to SimCLR****[6]****:** SimCLR utilizes negative samples (“dissimilarity”) to prevent convergence issues, whereas SimTrip is akin to “SimCLR without the negative samples, incorporating an additional augmented view instead”. SimCLR typically requires a large batch size to build the positive and negative samples for training an efficient model, demanding substantial computational resources. The SimCLR approach, as inferred from Chen and He [9]'s ablation study in Table 6, implies that neither stop-gradient features nor the additional prediction MLP yield benefits for SimCLR, suggesting that these elements address different optimization challenges than contrastive learning.

**Table:** 

SimCLR	-predictor	-predictor & -stop-grad

66.5	66.4	66.0

**Relation to BYOL****[8]****:** The SimTrip can be described as “BYOL without momentum encoder with an additional augmented view”. The momentum encoder in BYOL is considered to improve accuracy [9], but it is not essential to prevent collapse.

**Relation to SwAV****[11]****:** SimTrip is analogous to “SwAV without online clustering plus an additional augmented view”. We establish this comparison by adjusting certain aspects of SwAV. Firstly, SwAV's shared prototype layer can be integrated into the SimTrip's encoder. Secondly, while prototypes in SwAV are weight-normalized outside of the gradient propagation in [11], we perform full gradient computations as detailed in [53]. Thirdly, SwAV's similarity function is based on cross-entropy, but our method is based on negative cosine similarity.

Moreover, SwAV employs the Sinkhorn-Knopp (SK) transformation [54] on its target branch, which is also symmetrized [11]. The SK transformation, originating from online clustering [11], clusters the present batch while adhering to a balanced partition constraint, which helps to prevent collapsing. Our method does not use this transformation.

**Relation to SimSiam****[9]****:** Compared with SimSiam, we not only add an additional augmented view but also introduce a new triplet loss function TriLoss, instead of symmetrized loss. Moreover, SimTrip has undergone exhaustive experimentation in designing the architecture and the parameters of the projection and prediction MLPs, leading to more effective components of SimTrip as described in Fig. 2. By adding these modifications, our results are better than the SimSiam, as explained in the Sect. 4.

## 4 Experiments and results

The section outlines a comprehensive experimental evaluation designed to validate the effectiveness of the SimTrip model. In Sect. 4.1, we provide all the details of the experimental setup. In Sect. 4.2, we illustrate the results of linear evaluation with different datasets and compare them with other state-of-the-art methods. Next, we show the fine-tuning results with different numbers of labels for the different datasets and then compare the results with other leading methods in Sect. 4.3. Finally, we empirically study the behaviours of SimTrip and analyse the results of the ablation experiment in Sect. 4.4.

### 4.1 Experimental settings

**Fig. 7:**
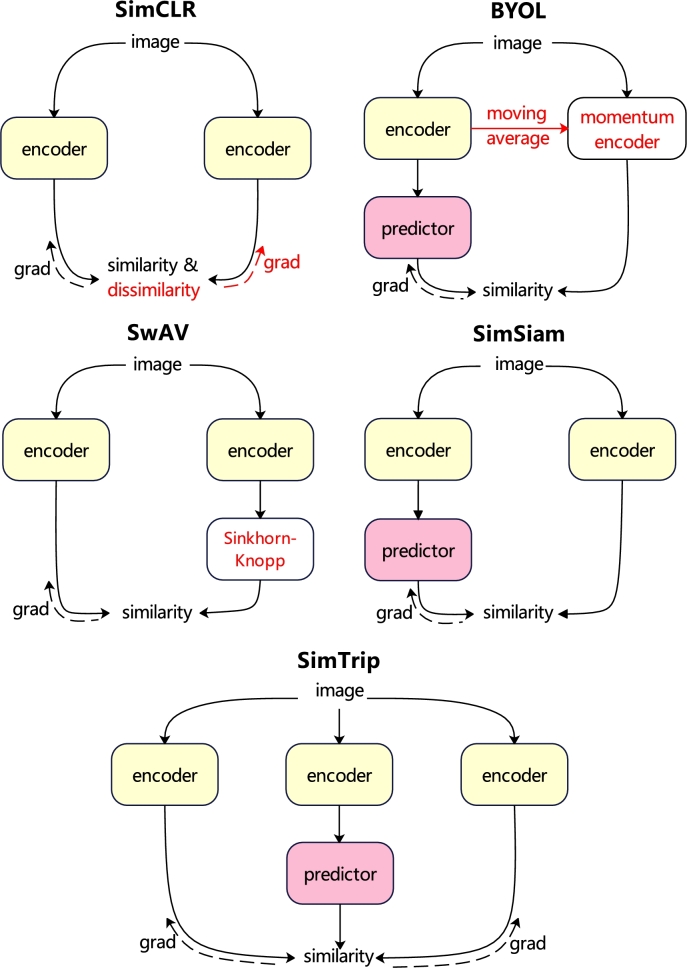
Four examples of different acute lymphoblastic leukaemia classes from ALL dataset: (**a**) Benign, (**b**) Early Pre-B, (**c**) Pre-B, and (**d**) Pro-B.

**Fig. 8:**
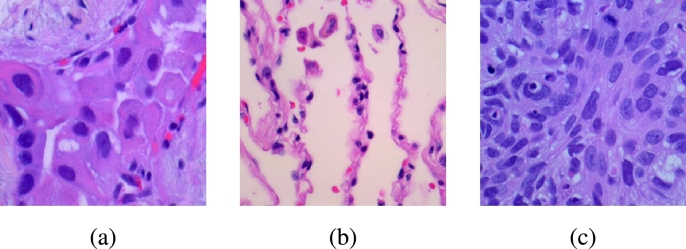
Three examples of different lung cancer classes from the LC25000 dataset: (**a**) lung adenocarcinoma, (**b**) lung benign, (**c**) lung squamous cell carcinoma.

We utilise two medical image datasets to evaluate the efficacy of the SimTrip model comprehensively: the Acute Lymphoblastic Leukemia image dataset (ALL) [36] and the Lung and Colon Cancer Histopathological Images dataset (LC25000) [35]. The ALL dataset consists of 3256 peripheral blood smear (PBS) images, dichotomising into benign and malignant categories. The benign category encompasses images with hematogenous, while the malignant category is further subdivided into three subclasses indicative of various stages of acute lymphoblastic leukaemia: Early Pre-B, Pre-B and Pro-B. In total, there are four classes, as shown in Fig. 7. The LC25000 dataset encompasses a collection of 25000 images, categorising five distinct histopathological types of lung and colon tissues: benign lung tissue, lung adenocarcinoma, lung squamous cell carcinoma, colon adenocarcinoma, and benign colon tissue, as shown in Fig. 8.

In the training process, the dataset splits into training and testing sets following an 80:20 ratio. Subsequently, the training dataset splits with different labelled ratios into two sub-training datasets: unlabelled and labelled training datasets. Our experiments run with an NVIDIA RTX 3090 GPU with 24 GB of RAM and an Xeon CPU with 80 GB of RAM. The code is implemented via the Keras [37] framework, augmented with the scikit-learn [38] library for additional data processing and analytical capabilities. The hyperparameters of SimTrip are delineated in Table 1.

**Table 1:** Hyper-parameters of experiment.

Name	Value
image size	256 × 256 × 3
epochs	200
batch size	256
projection dimension	8192
latent dimension	2048
learning rate	0.05

To substantiate the robustness of SimTrip, a series of proxy tasks are deployed, containing linear evaluation and fine-tuning across different medical image datasets. The performance of SimTrip is not only benchmarked against the fully supervised state-of-the-art methods but also compared with other leading self-supervised learning-based methods, including SimCLR [6], SimSiam [9] and UKSSL [51]. These methods are retrained on the same medical datasets to facilitate a direct comparative analysis.

The evaluation metrics employed to gauge the performance encompass accuracy, precision, recall and the F1-score. These metrics are derived using Eqn. (18) to (21), respectively. Here, TP denotes True Positives, FP signifies False Positives, FN represents False Negatives, and TN stands for True Negatives, providing a comprehensive statistical measure of the model's performance.

(18) $$accuracy=\frac{TP+TN}{TP+FP+FN+TN},$$

(19) $$recall=\frac{TP}{TP+FN},$$

(20) $$precision=\frac{TP}{TP+FP},$$

(21) $$F1\text{-}score=\frac{2\times precision\times recall}{precision+recall}.$$

### 4.2 Linear evaluation

In this section, we present a comprehensive evaluation of the SimTrip through linear evaluation on the ALL and LC25000 datasets. Moreover, we compare the performance of SimTrip with other state-of-the-art methods with different data supervision: supervised, semi-supervised and unsupervised approaches. This comparative study serves to validate the efficacy of our proposed SimTrip and to underscore its superior performance relative to extant state-of-the-art methods.

Table 2 elucidates the performance of SimTrip on the ALL dataset, where it achieves a precision of 99.7%, a recall of 99.9%, an F1-score of 99.8% and an accuracy of 99.8%. When benchmarked against prevailing supervised methods, SimTrip secures the highest precision. Its recall equates to the apex recall of 99.9% reported by Basymeleh et al. [41], while its F1-score is 0.5% higher than the DSCNet [40], and the accuracy also is the highest one which is greater than the Modified DenseNet201 [42] by 0.2%. Furthermore, we replicate several unsupervised and semi-supervised methods, such as SimCLR [6], SimSiam [9] and UKSSL [51], to draw comparisons with SimTrip. SimTrip manifests substantial gains over SimCLR [6], with improvements of 42.2% in precision, 55.2% in recall, 57.2% in F1-score, and 54.1% in accuracy. Relative to SimSiam [9], SimTrip exhibits marginal enhancements of 0.6% across all metrics, and it also outperforms UKSSL [51] by 2.1%, 2.4%, 2.2%, and 1.9% in terms of precision, recall, F1-score and accuracy.

**Table 2:** Linear evaluation results compared with state-of-the-art methods on the ALL dataset.

Author	Method	Precision	Recall	F1-score	Accuracy
Abdullah et al. [39]	DenseNet + MC-Dropout (supervised)	-	-	-	97.7%
	DenseNet + VI	-	-	-	94.4%
Kaur et al. [40]	DSCNet (supervised)	-	99.7%	99.3%	99.3%
Basymeleh et al. [41]	VGG16 (supervised)	-	99.9%	-	98.4%
Sajon et al. [42]	Modified DenseNet201 (supervised)	-	-	-	99.6%
Gokulkrishnan et al. [43]	Transfer Learning (supervised)	-	-	-	98.6%
Subramanian et al. [50]	VGG16 (supervised)	-	-	-	75.8%
	VGG19 (supervised)	-	-	-	76.3%
	DenseNet201 (supervised)	-	-	-	79.8%
	MobileNetV3-Small (supervised)	-	-	-	84.5%
	MobileNetV3-Large (supervised)	-	-	-	81.6%

Chen et al. [6]	SimCLR (unsupervised, repro.)	57.5%	44.7%	42.6%	45.7%
Chen and He [9]	SimSiam (unsupervised, repro.)	99.1%	99.3%	99.2%	99.2%
Ren et al. [51]	UKSSL (semi-supervised, repro.)	97.6%	97.5%	97.6%	97.9%

**Ours**	**SimTrip**	**99.7%**	**99.9%**	**99.8%**	**99.8%**

In Table 3, we report the linear evaluation results of SimTrip on the LC25000 dataset, where it attains 99.1% across precision, recall, F1-score and accuracy. Against leading supervised methods, SimTrip reaches the highest performance, outstripping the CNN proposed by Hatuwal and Thapa [47] by 1.8% in precision, recall and F1-score, 1.9% in accuracy, and outperforming the Shallow-CNN [48] by 1.3% in accuracy. When compared with the reproduced unsupervised and semi-supervised methods, including SimCLR [6], SimSiam [9], and UKSSL [51], SimTrip maintains the highest performance. It improves by 29.7%, 36.4%, 36.0%, and 36.4% in precision, recall, F1-score and accuracy with SimCLR [6]. The result of SimSiam [9] is the same as our method. Against UKSSL [51], our method shows an incremental advantage of 0.8% across all measured metrics.

**Table 3:** Linear evaluation results compared with state-of-the-art methods on the LC25000 dataset.

Author	Method	Precision	Recall	F1-score	Accuracy
Bukhari et al. [44]	RESNET50 (supervised)	95.7%	81.8%	96.2%	93.9%
	RESNET18 (supervised)	93.0%	84.2%	95.7%	93.0%
	RESNET34 (supervised)	93.0%	80.9%	95.7%	93.0%
Phankokkruad [45]	Ensemble (supervised)	92.0%	91.0%	91.0%	91.0%
	ResNet50V2 (supervised)	91.0%	90.0%	90.0%	90.0%
Hlavcheva et al. [46]	CNN-D (supervised)	-	-	-	94.6%
Hatuwal and Thapa [47]	CNN (supervised)	97.3%	97.3%	97.3%	97.2%
Mangal et al. [48]	Shallow-CNN (supervised)	-	-	-	97.8%
Masud et al. [49]	DL-based CNN (supervised)	96.3%	96.3%	96.3%	96.3%

Chen et al. [6]	SimCLR (unsupervised, repro.)	69.4%	62.7%	63.1%	62.7%
Chen and He [9]	SimSiam (unsupervised, repro.)	**99.1%**	**99.1%**	**99.1%**	**99.1%**
Ren et al. [51]	UKSSL (semi-supervised, repro.)	98.3%	98.3%	98.3%	98.3%

**Ours**	**SimTrip**	**99.1%**	**99.1%**	**99.1%**	**99.1%**

In summary, linear evaluation results demonstrate that our SimTrip achieves the pinnacle of performance across both the ALL and LC25000 datasets when compared to a spectrum of state-of-the-art methods. This confirms that SimTrip not only excels in fully supervised settings but also demonstrates formidable results under weakly supervised conditions, thereby endorsing the versatility and robustness of our proposed approach.

### 4.3 Fine-tuning results with different numbers of labels

Table 4 and Table 5 present a comprehensive evaluation of SimTrip's performance through the fine-tuning of a linear classifier across varying label availability by ALL and LC25000 datasets. We reproduce the approaches of SimCLR [6], SimSiam [9], and UKSSL [51], assessing them against different label proportions (1%, 10%, 25%, and 50%) and different batch size (256 and 500) on the ALL and LC25000 datasets and then comparing the results of these methods with SimTrip.

**Table 4:** Fine-tuning results compared with state-of-the-art methods on the ALL dataset.

Author	Method	Batch Size	Labelled Ratio	Precision	Recall	F1-score	Accuracy
Chen et al. [6]	SimCLR (repro.)	256	1%	54.2%	50.6%	45.8%	50.6%
			10%	62.1%	52.0%	50.7%	55.2%
			25%	58.3%	47.1%	43.7%	47.1%
			50%	54.6%	43.6%	38.3%	42.3%
		500	1%	53.8%	40.1%	35.6%	41.0%
			10%	56.7%	44.8%	42.5%	46.0%
			25%	56.7%	45.3%	43.2%	46.3%
			50%	68.1%	40.3%	37.4%	43.9%

Chen and He [9]	SimSiam (repro.)	256	1%	75.4%	73.2%	73.9%	75.9%
			10%	91.2%	92.4%	91.5%	92.0%
			25%	97.4%	98.2%	97.8%	98.0%
			50%	98.8%	98.9%	98.9%	98.9%
		500	1%	81.9%	77.1%	77.5%	79.7%
			10%	94.7%	**94.6%**	94.6%	95.1%
			25%	97.0%	97.3%	97.2%	97.4%
			50%	98.2%	98.3%	98.2%	98.3%

Ren et al. [51]	UKSSL (repro.)	256	1%	6.9%	25.0%	10.8%	27.6%
			10%	82.1%	81.1%	81.4%	84.7%
			25%	89.2%	86.2%	83.6%	86.2%
			50%	89.8%	86.5%	84.2%	86.5%
		500	1%	59.1%	68.6%	63.4%	76.4%
			10%	82.4%	80.9%	81.4%	82.8%
			25%	83.4%	82.2%	82.7%	84.7%
			50%	97.0%	93.0%	94.4%	95.4%

**Ours**	**SimTrip**	256	1%	**88.6%**	**85.8%**	**86.7%**	**88.0%**
			10%	**95.5%**	94.3%	**94.9%**	**95.2%**
			25%	**99.5%**	**99.6%**	**99.5%**	**99.5%**
			50%	**99.6%**	**99.7%**	**99.7%**	**99.7%**

**Table 5:** Fine-tuning results compared with state-of-the-art methods on the LC25000 dataset.

Author	Method	Batch Size	Labelled Ratio	Precision	Recall	F1-score	Accuracy
Chen et al. [6]	SimCLR (repro.)	256	1%	67.1%	59.0%	59.4%	59.3%
			10%	67.0%	59.3%	58.6%	59.3%
			25%	63.5%	56.5%	55.6%	55.8%
			50%	68.6%	61.9%	62.6%	61.8%
		500	1%	64.5%	56.1%	54.7%	56.1%
			10%	66.3%	59.0%	58.6%	59.0%
			25%	66.7%	59.2%	58.9%	59.2%
			50%	67.2%	62.1%	62.4%	62.1%

Chen and He [9]	SimSiam (repro.)	256	1%	95.8%	95.7%	95.7%	95.6%
			10%	97.0%	97.0%	97.0%	97.0%
			25%	99.0%	99.0%	99.0%	99.0%
			50%	**99.1%**	**99.1%**	**99.1%**	**99.1%**
		500	1%	94.5%	94.6%	94.5%	94.6%
			10%	97.6%	97.6%	97.6%	97.6%
			25%	98.7%	98.7%	98.7%	98.7%
			50%	98.9%	98.9%	98.9%	98.9%

Ren et al. [51]	UKSSL (repro.)	256	1%	4.3%	20.0%	7.1%	21.4%
			10%	4.0%	20.0%	6.7%	20.0%
			25%	92.1%	92.4%	92.0%	91.9%
			50%	96.5%	96.6%	96.5%	96.5%
		500	1%	45.5%	54.6%	43.8%	55.5%
			10%	91.5%	90.8%	90.7%	90.6%
			25%	96.3%	96.3%	96.3%	96.3%
			50%	98.9%	98.9%	98.9%	98.9%

**Ours**	**SimTrip**	256	1%	**95.9%**	**96.0%**	**95.9%**	**96.0%**
			10%	**98.5%**	**98.5%**	**98.5%**	**98.5%**
			25%	**99.1%**	**99.1%**	**99.1%**	**99.1%**
			50%	**99.1**%	**99.1%**	**99.1%**	**99.1%**

Table 4 delineates the comparative performance metrics of SimTrip against other leading-edge methods on the ALL dataset. At a labelled ratio of 1%, SimTrip achieves superior precision (88.6%), recall (85.8%), F1-score (86.7%) and accuracy (88.0%). This represents a significant improvement over other methods, with at least a 6.7% increase in precision, 8.7% in recall, 9.2% in F1-score, 8.3% in accuracy with SimSiam [9] trained with batch size of 500, and at least 11.6% increase in accuracy with UKSSL [51]. When changing the labelled ratio to 10%, our SimTrip also gets the better performance and improves at least 0.8% in precision, 0.3% in F1-score and 0.1% in accuracy with SimSiam [9] trained with a batch size of 500. With the labelled ratio of 25%, SimTrip outperforms other methods by at least 2.1%, 1.4%, 1.7%, and 1.5% in terms of precision, recall, F1-score and accuracy. Similarly, the SimTrip also gets the highest performance with the labelled ratio of 50%, which obtains 99.6% of precision, 99.7% of recall, F1-score and accuracy, respectively. It outperforms other methods by at least 0.8% in precision, recall, F1-score and accuracy, individually. Regarding the batch size, although SimTrip deploys with a batch size of 256, it still performs well compared with other methods deploy with various batch sizes.

As shown in Table 5, we compare the fine-tuning results of SimTrip with other state-of-the-art methods on the LC25000 dataset. The overall performance of SimTrip is also the best compared with other methods under the different labelled ratios and batch sizes. It needs to be mentioned that when the labelled ratio is 50%, the SimTrip gets the same results as the SimSiam [9] with precision, recall, F1-score and accuracy all at 99.1%. But the SimTrip still gets the best results compared with SimCLR [6], SimSiam [9] and UKSSL [51] under the different settings of labelled ratios and batch sizes.

To conclude, the fine-tuning results demonstrate that SimTrip not only excels with minimal labelled data but also exhibits the potential for significantly enhanced performance with increased labelled proportions. This comparative analysis substantiates SimTrip's efficacy across both ALL and LC25000 datasets and endorses its robustness against a spectrum of unsupervised and semi-supervised learning paradigms. Also, the batch size used in SimTrip is less than other methods, which is an advantage to run experiments on lower computational power.

### 4.4 Empirical study

We verify the effectiveness of each component in SimTrip, and the results are shown in Table 6, Table 7. This ablation study employs a linear evaluation task on the 1% and 10% labelled ALL dataset, examining five distinct configurations that represent various combinations of components. Here, we take the data from Table 6 to make a comparison, because the conclusion summarised from Table 7 is the same as Table 6.

**Table 6:** Ablation study of SimTrip on the ALL dataset (1%).

	Baseline	+Image Augmentations	+Projection MLP	+Prediction MLP	SimTrip
Accuracy (%)	83.6	84.2	84.0	85.7	88.0
Precision (%)	86.4	83.2	86.8	83.0	88.6
Recall (%)	79.6	78.6	76.8	84.7	85.8
F1-score (%)	80.0	78.9	77.3	83.0	86.7

**Table 7:** Ablation study of SimTrip on the ALL dataset (10%).

	Baseline	+Image Augmentations	+Projection MLP	+Prediction MLP	SimTrip
Accuracy (%)	93.5	91.4	93.1	94.3	95.2
Precision (%)	93.8	91.8	92.7	92.8	95.5
Recall (%)	92.2	88.9	92.2	94.1	94.3
F1-score (%)	92.9	89.8	92.4	93.3	94.9

**Baseline:** Baseline removes the image augmentation module, projection MLP and prediction MLP. It achieves an accuracy of 83.6%, a precision of 86.4%, a recall of 79.6% and an F1-score of 80.0%.

**+Image Augmentations:** It originates from the baseline with the image augmentation module without projection MLP and prediction MLP. This setting achieves 84.2% in accuracy, 83.2% in precision, 78.6% in recall, and 78.9% in F1-score.

**+Projection MLP:** It removes the image augmentation module and prediction MLP, and remains the projection MLP based on the baseline. This setting obtains results of 84.0% (accuracy), 86.8% (precision), 76.8% (recall) and 77.3% (F1-score).

**+Prediction MLP:** This setting, building upon the baseline by integrating prediction MLP excluding image augmentation module and projection MLP, records 85.7% of accuracy, 83.0% of precision, 84.7% of recall and 83.0% of F1-score.

**SimTrip:** The complete model, which encompasses all the components mentioned above, and the results are reported in Table 4.

The ablation study reveals that image augmentation module are instrumental in generating variant views of the same image, thereby enriching the model's capacity for positive sample extraction and feature discernment from the dataset. Projection MLP and prediction MLP emerge as crucial elements of the method, facilitating solving the model collapse problem. As evidenced in Table 6, the integration of all components within the SimTrip culminates in the most auspicious outcomes, with each element independently validating its utility and their collective interaction yielding efficacious cooperation.

## 5 Discussion

The preceding evaluation substantiates the versatility and superior performance of our SimTrip across various downstream tasks, including linear evaluation and fine-tuning, where it outpaces existing state-of-the-art methods. Notably, SimTrip achieves the best performance comparable to supervised methods while requiring only a subset of labelled data for training. Moreover, unlike other contrastive learning approaches, SimTrip obviates the need for a memory bank [5] to store positive and negative samples, as well as the process of constructing negative samples, which is a requirement exemplified by SimCLR [6], and it needs to compute the loss between the positive and negative pairs. Compared with SimSiam [9], our SimTrip can reach higher performance, as we presented in the Sect. 4.

A distinctive advantage of SimTrip is its efficiency with smaller batch sizes. The triplet loss used in SimTrip makes it works more efficiently, even in small batch sizes. In our experiments, we employ a batch size of 256, drawing on the triplet loss's ability to generate three views from a single sample. This contrasts with SimSiam [9], which relies on two views for loss computation, suggesting that SimTrip could maintain efficacy even with reduced batch size. The smaller batch size requirement significantly diminishes computational demands, enabling training on a single RTX3090 GPU, whereas models like SimCLR [6] and UKSSL [51] are difficult to train with small batch size, due to their reliance on constructing positive and negative pairs within each batch, necessitating larger batch sizes for optimal model training. When comparing the performance of SimTrip with other models, although the results shown in Table 4, Table 5 prove that the SimCLR [6] and UKSSL [51] are performed well on large batch size, the proposed SimTrip still get better performance with smaller batch size.

There are a few limitations or challenges of our method. The computational resources currently available to us preclude the execution of transfer learning tasks. Typically, such tasks would involve pre-training on a comprehensive dataset, such as ImageNet [52], followed by fine-tuning on the target dataset. However, the ImageNet includes 14197122 annotated images, and the datasets used in our experiments only have 3256 (ALL) and 25000 (LC25000) images. It is time-consuming and costly to train a model with the ImageNet dataset, and we will explore it in future research. Additionally, the present performance is limited by the complexity of the proxy tasks. For instance, in our experiments reported in Sect. 4, we only use a single linear layer for fine-tuning. The more complex architecture of classifiers can improve the performance of the method, as seen in the UKSSL [51]. They reported that the implementation of the well-designed UKMLP classifier achieves better performance than the single linear layer classifier. Their ablation study assumes that the complex classifier performs better than the simple classifier. In future, we will employ SimTrip as a feature extractor and pair it with more complex classifiers to enhance performance in a semi-supervised learning paradigm. These limitations constrain the higher performance of our proposed method, and some misclassified images still exist when applying our method. These misclassified images can be further reduced by embedding complex encoder backbones and replacing the linear classifier with other multi-layer classifiers.

The potential applications in practical scenarios of our method are manifold, spanning classification, object detection and segmentation. The benefits of contrastive learning within SimTrip are particularly salient in domains where only partially labelled datasets are available, such as medical image analysis. Moreover, its suitability for applications where computational efficiency or labelling cost savings are paramount makes it an attractive option. SimTrip could also serve as an unsupervised feature extractor that, when integrated with supervised models, facilitates the construction of semi-supervised frameworks.

## 6 Conclusion

This paper proposed a new contrastive learning method SimTrip, which excels in tasks requiring minimal labelled data, outperforming state-of-the-art methods. By leveraging the proposed TriLoss loss and facilitating training with smaller batch sizes, it operates efficiently on computational power-limited hardware, expanding access to advanced machine learning techniques. Despite limitations in computational resources and proxy task complexity, SimTrip's potential in practical applications, particularly where labelled data is scarce, is substantial. Future research will focus on enhancing its transfer learning capabilities and combining it with complex classifiers to bolster its performance in a semi-supervised learning framework.

## Declaration of Competing Interest

The authors hereby declare that there are no conflicts of interest to report. This declaration encompasses all potential areas of conflict, including financial, personal, and professional relationships, that could be perceived as influencing the research process, the interpretation of findings, or the conclusions drawn from this study.

## Appendix A Supplementary material

MMC User Guide for SimTrip Classification System. MMC
