# Supplementary material for: Exploring simple triplet representation learning
Source: Comput Struct Biotechnol J. 2024 Apr 12;23:1510–21. doi: 10.1016/j.csbj.2024.04.004 (PMC11021836; doi:10.1016/j.csbj.2024.04.004)
Supplement: MMC — User Guide for SimTrip Classification System. [file mmc1.pdf]

User Guide for SimTrip Classification System

1. Introduction

Welcome to the SimTrip Classification System, a cutting-edge web application designed to classify lung cancer images with precision and ease. This guide will walk you through the process of using the system, from uploading your images to interpreting the classification results.

2.Getting Started

2.1 Running Online

Open the browser, and paste the address: http://43.131.9.159:5000/ (note: sometimes it loads very slow, please try it again later.)

Download the test images from:
https://github.com/JerryRollingUp/SimTripSystem/tree/master/example_imgs

Follow the steps from Section 2.2.3.

2.2 System Requirements

To use the SimTrip Classification System, ensure you have access to a modern web browser such as Google Chrome, Mozilla Firefox, Safari, or Microsoft Edge. The system is accessible on desktops, laptops, with internet connectivity.

2.2.1 Configuration of systems

The configuration of this system is summarised in the 'readme.md' file, please follow it to config this system.

2.2.2 Accessing the System

- When you finished the previous steps, there is an address shown in the terminal, show as follows:

```
(tf) (base) Jerry@Jerry SimTripSystem % python main.py
2024-03-14 22:55:43.302108: I tensorflow/core/platform/cpu_feature_guard.cc:182] This TensorFlow binary is optimized to use available CPU instructions in performance-critical operations.
To enable the following instructions: AVX2 FMA, in other operations, rebuild TensorFlow with the appropriate compiler flags.
 * Serving Flask app 'main'
 * Debug mode: on
WARNING: This is a development server. Do not use it in a production deployment. Use a production WSGI server instead.
 * Running on http://127.0.0.1:5000
Press CTRL+C to quit
 * Restarting with stat
 * Debugger is active!
 * Debugger PIN: 988-037-519
```

- Copy the address and copy to your web browser (In this tutorial the address is: http://127.0.0.1:5000/), the web page looks like as follows:

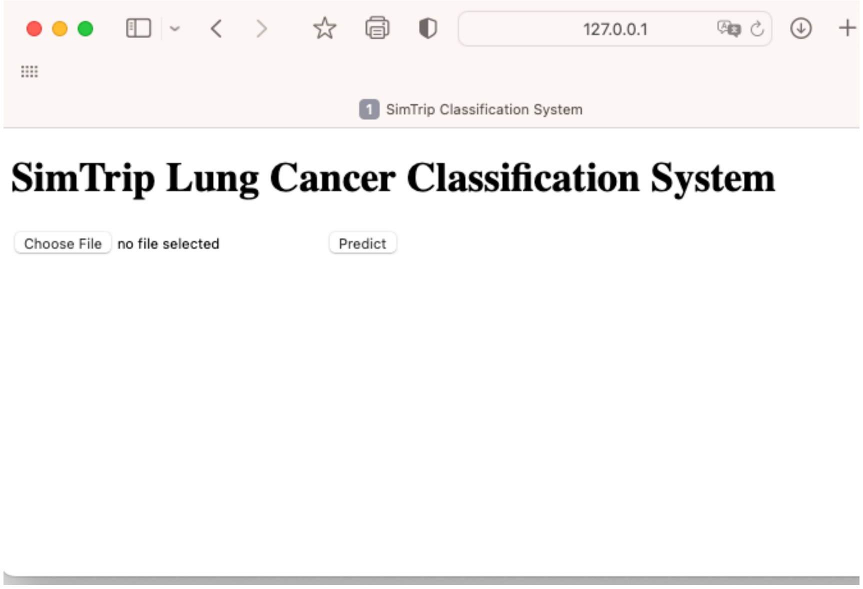

2.2.3 Prediction

- There are two buttons: 'Choose File' and 'Predict'. You need to select a lung cancer image by clicking 'Choose File' from your local drive or select some examples included in this project. Here, we use a lung cancer image from this project, the lung cancer class of it is 'Lung adenocarcinoma'

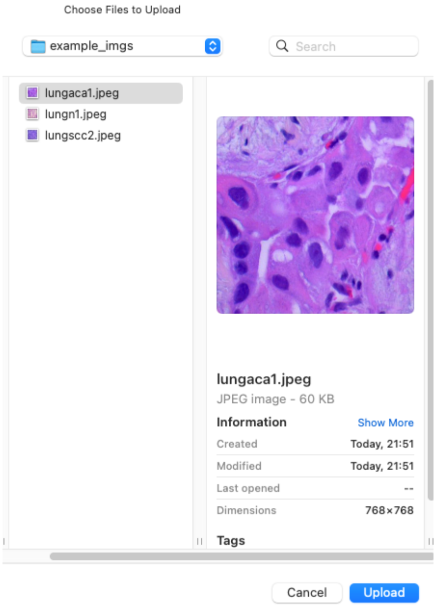

- When you upload the image, the system should look like this:

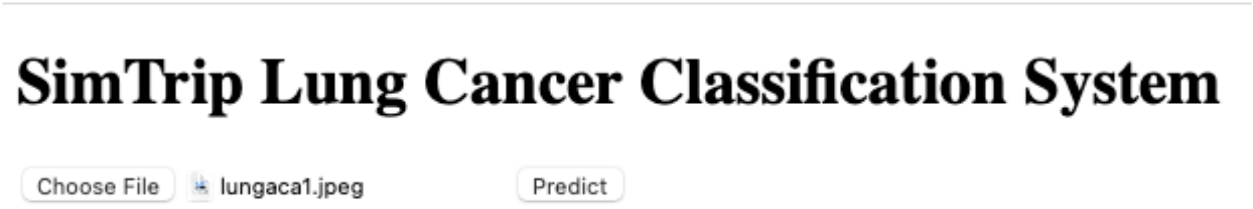

- Predict the results by clicking ‘Predict’. And the system will return the results! The predicted result is the same as the lung cancer type of the image.

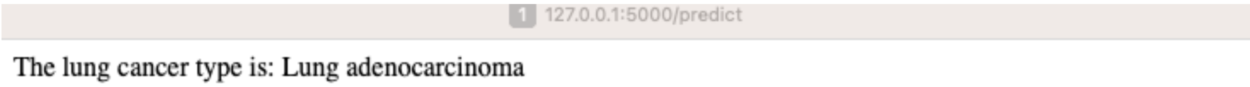

- You can go back to previous web page and submit a new image to predict the result.

Tips for Best Results

- Image Quality: For best results, ensure that the images are clear and focused. Blurry or low-resolution images may affect the accuracy of the classification.
- Image Format: Only upload images in JPEG or PNG format. Other formats are not supported and will result in an error.
- Multiple Images: If classifying multiple images, consider uploading them one at a time for more detailed results.

Troubleshooting

- Upload Errors: If you encounter issues uploading images, ensure that the files are not corrupted and are in the supported format.
- Processing Delays: High system demand may occasionally result in processing delays. If your results are taking longer than expected, please be patient or try uploading your image(s) again later.

Support

If you need assistance or have any questions, please contact our support team at zr41@leicester.ac.uk. We are here to help you with any issues or inquiries you may have.

Conclusion

Thank you for choosing the SimTrip Classification System for your lung cancer image classification needs. We are committed to providing you with a reliable and user-friendly tool to assist in the diagnosis and study of lung cancer. Your feedback is valuable to us as we strive to improve and enhance the system.
